# List of Diseases at the Bath City Dispensary, from August 1, to September 1, 1802

**Published:** 1802-10-01

**Authors:** 


*List of Diseases at the Bath City Dispensary, from August* 1, *to September* 1, 1802.

| | | | |
|---|---|---|---|
| Angina | 2 | Pulmonary Complaints | 8 |
| Arthritis | 1 | Phthisical | 6 |
| Anasarca | 4 | Phthisis Scrophulosa | 1 |
| Asthenia | 1 | Peripneumonia Notha | 1 |
| Bilious Complaints | 4 | Paralysis | 3 |
| Colica | 2 | Psora | 1 |
| Dysenteria | 2 | Rheumatismus Acutus | 11 |
| Diarrhœa | 1 | Rubeola | 2 |
| Erysipelas | 2 | Synochus Biliosa | 17 |
| Enteritis | 1 | ——— Quotidiana | 1 |
| Eruptiones | 2 | Syphilis | 2 |
| Febris | 4 | Scrofula | 1 |
| ——— Intermittens Quotidiana | 2 | Strain | 3 |
| Gastrodynia | 3 | Typhus Mitior | 4 |
| Hæmoptysis | 3 | Tussis | 1 |
| Hæmatemesis | 1 | Tinea Capitis | 3 |
| Hepatitis Chronica | 1 | Variola | 4 |
| Leucorrhœa | 2 | Vertigo | 1 |
| Lepra | 2 | Vermes | 2 |
| Lithiasis | 1 | Ulcerated Legs | 5 |
| Menorrhagia | 1 | Ulcers | 3 |
| Nephritic Complaints | 2 | | |
| Ophthalmia | 1 | Total | 125 |

